# Cases of Epulis

**Published:** 1890-04

**Authors:** J. F. Colyer


ARTICLE VI.

### CASES OF EPULIS.

BY J. F. COLYER, M. R. C. S., L. D. S., ETC.

J. J., aet. 30, applied for advice concerning a small growth in mouth. Examination revealed a small, painless fibrous tumour in the region of the right upper lateral and canine teeth, which had been growing during the previous three years.

*Treatment*:—Cocaine (gr. ½ dissolved in 7m. distilled water) was injected, and the growth removed by means of a small scalpel, the bone between the teeth from which the growth sprung being cut away by small enamel chisels and coarse cut burrs on the dental engine.

The wound thus made was carefully syringed with carbolic acid (1 in 40), and crystals of chloride of zinc applied. The patient being directed to use the following as a mouth wash:—

R Acidi Carbolici (glacialis) - } āā ʒ ii

Liq. Potassæ, - - - }

Aquam, - - - ad. ℥ ii

*m* Fiat Lotio.

One teaspoonful in half a tumbler of water to be used frequently.

The wound healed in the course of fourteen days. When last seen, which was eighteen months since removal, there were no signs of any recurrence.

CASE II.

Miss F. B., æt. 13, applied at the hospital in February, 1888, with regard to a small growth in the region of the right lower canine and lateral.

The history was briefly as follows:—About five years previously, patient noticed a swelling which continued slowly to increase in size; this persisted for nearly three years, when she went to a doctor who removed it, but it recommenced to grow immediately afterwards, and was again removed a year previously to her applying at the hospital.

When seen, the growth appeared to be springing from the septum between the teeth named. Mr. Claude Rogers, under whose care the patient was, recommended the removal of the lateral, and the cutting away of bone around the canine.

This operation was carried out under ether, the bone being removed in the same manner as stated in the previous case and the same after treatment adopted.

When last seen, which is now nearly two years since the operation, there appeared no signs of any recurrence.

*Remarks.*—These two cases which in themselves present nothing remarkable, are, however, useful, since they show the two courses which are open in the treatment of epulis, viz. (1), by removing the growth and retaining the tooth, and (2), by removing the growth and tooth as well. Many hold that for the successful treatment of epulis the tooth should be removed in all cases; that this is not absolutely necessary I feel sure, the majority of cases met with in dental practice being able to be treated as successfully by retaining the tooth, as by removing it.

The lines of treatment should, I think, be, to first remove the growth and retain the tooth, then if this fails, resort to the extraction of the tooth as well.

If it is decided to retain the tooth, then success depends on the thoroughness with which the bone is removed, and this can always be accomplished with enamel chisels and coarse cut conical burrs. With regard to the treatment of the wound caused by the removal nothing seems to promote the formation of healthy granulations better than chloride of zinc, and this should be applied to the wound at intervals of about three to four days, (two applications being generally sufficient), and during those periods the mouth kept clean by means of an antiseptic mouth wash. *London Dental Record.*

